# Improving the Learning Experience through Evidence-based Education

**DOI:** 10.5811/westjem.2018.10.41320

**Published:** 2018-11-13

**Authors:** Jeffrey N. Love, Anne M. Messman, Chris Merritt

**Affiliations:** * MedStar Georgetown University Hospital, MedStar Washington Hospital Center, Department of Emergency Medicine, Washington, District of Columbia; † Wayne State University, Sinai-Grace Hospital/Detroit Receiving Hospital, Department of Emergency Medicine, Detroit, Michigan; ‡ Alpert Medical School of Brown University, Rhode Island Hospital/Hasbro Children’s Hospital, Department of Pediatric Emergency Medicine, Providence, Rhode Island

Our learners invest a significant portion of their time, energy and financial future to achieve the goal of becoming physicians. In turn, society expects physicians who are well trained and prepared for a career that includes lifelong learning. What both our learners and ultimately their patients get in return too often falls short of what should be a first-rate experience. With the many hours spent in lectures, how often has the information conveyed changed your practice? During your education, how often have you felt like an active participant whose specific needs were being addressed? To what extent has lifelong learning been modeled for you throughout your education? These and many other questions regarding our own educational experiences as physicians can and should be addressed by the practice of ***evidence-based education***.

For generations, physicians in training have been taught by educators who were content experts but had little, if any, formal training in education. This includes many of today’s education leaders who develop their local culture and design the educational experiences. One might argue that we all turned out all right as physicians in a less-than-ideal educational environment, but today’s standard demands something more. There is a general move toward competency-based assessment and longitudinal, clinically relevant curricula.^1^–^2^ In this environment where the status quo is no longer sufficient, there is an opportunity to develop experiences based on evidence that provide a high-quality education supporting both our learners in the pursuit of their calling and our mission as educators.

Indeed, there is an increasing emphasis on applying learning theory and best practices in our educational endeavors. This can be seen in the development of educator tracks in medical school and residency, education fellowships, faculty development programs, and advanced degrees in education.^3^–^5^ There is much that can be done to maximize our effectiveness as educators. Toward that end, we advocate for a concerted effort to practice evidence-based education by faculty whenever presented with an educational endeavor. Formal advanced training in education is not required to practice evidence-based education, just as formal training in statistics and epidemiology may not be required for evidence-based clinical practice, but some understanding of educational theory and best practice is critical. Although seeking relevant resources for an educational task, such as current concepts, guidelines, and research can be daunting, the benefit of such an approach to the educator and learner are without measure (Table 1).

Central to success in any educational initiative is an understanding of the importance of ***conceptual frameworks*** (e.g., theories, best practices, models). How to best accomplish an educational goal can be viewed from a number of perspectives; the conceptual framework selected highlights the authors’ choice of theory or best practice most likely to accomplish their intended goal.^6^ Once selected, this framework becomes the basis for decision-making regarding the design of the education experience and outcomes to be measured. Though one dominant conceptual framework is generally selected for any given initiative, a number of additional theories or best practices may be relevant to the design of the experience. From an academic perspective, the lack of a conceptual framework is a common reason for journals to reject scholarly submissions.^7^,^8^

Educational initiatives, whether a presentation, workshop, rotation, curriculum or program, should include an early determination of the outcomes to be measured demonstrating the value or relative benefit of the initiative. Generally these outcomes reflect the goals of important stakeholders (e.g., learners, teachers, patients, administration). A presentation generally has relatively simple, straightforward outcomes while multifaceted, large-scale initiatives or those intended for scholarly submission require more substantive, objective outcomes. Common means of determining or evaluating appropriate outcomes include ***Kirkpatrick’s hierarchy***,^9^ ***Bloom’s taxonomy***,^10^ ***Miller’s pyramid***,^11^ ***Moore’s outcomes***,^12^ ***and the logic model***.^13^ Although questionnaires^14^ and assessment methods such as workplace-based assessments (i.e., clinical competency committees, Standardized Letters of Evaluation)^15^ don’t provide outcome measures that can be assessed in the same manner, it is no less important that they are based on psychometrically-sound principles in order to perform in the manner intended with the added potential to serve as scholarship.

Several examples from the authors’ own experience are provided to demonstrate and clarify the principles discussed:

## EXAMPLE 1

A few years after completing residency, this author took a job at a new institution as an assistant program director. It seemed that while the residents were very enthusiastic and eager to learn, they were regularly faced with a dilemma: whether or not to complete their weekly reading assignment. Since all residents were given identical weekly reading assignments, the topics were not always pertinent to what they were doing clinically. As an example, a resident could be on an obstetrics and gynecology rotation but the assigned reading could be on trauma. The resident often had to choose between reading about their patients to prepare for their clinical work, or completing the assigned reading for the week.

As an enthusiastic new faculty member, this author saw a problem and wanted to fix it. The goal was to create a system where everyone was on their own unique reading schedule, and their reading assignments would be coupled with their clinical work. Tempted to create a new reading curriculum and immediately implement it, the author recalled the prior teaching of medical education mentors: one must be deliberate about the changes made and should study the effects of those changes.

The concept of pairing a resident’s clinical work with their reading assignments is supported by ***Kolb’s theory of experiential learning***.^16^ Kolb argued that effective learning occurs as learners’ cycle through four stages: concrete experience, active experimentation, reflective observation, and abstract conceptualization. In the case of the reading curriculum, the plan was for the residents to have the concrete experience of readings relevant to what they were experiencing clinically, allowing them to actively experiment and reflect on the application of this newly acquired knowledge. Kolb’s theory suggests that this process would lead to more effective learning, which was the goal in changing the way reading was assigned to the residents.

In addition to ensuring that the curricular intervention was grounded in educational theory, the author was deliberate in her intention to evaluate the changes made. The Kirkpatrick outcome levels were very helpful in guiding the project toward these evaluation goals.^9^ To begin with, level 1 outcomes were evaluated having to do with the learner’s satisfaction with the curricular change. To evaluate this, the residents reported their level of satisfaction with the curriculum both before and after the curricular intervention. Level 2 outcomes measure the learner’s acquisition of knowledge. The plan was to improve the learners’ acquisition of knowledge by improving their reading schedule. Objectively measuring this outcome will be a long-term goal, perhaps accomplished by evaluating changes in in-training examination scores or some other objective test of knowledge. Level 3 outcomes have to do with changes in learners’ behavior. Consequently, the amount of time residents spent reading before and after the intervention was evaluated. It was found that they spent significantly more time reading after the intervention; thus, their behavior (i.e., motivation to read) appeared to have changed. Although levels 1 and 3 outcomes were measured, they were both subjective in that they were self-reported by the residents. As a curricular innovation based on education theory, the author was able to publish her work based on these preliminary findings.^17^ There are plans in place for more rigorous, objective outcomes to evaluate the value of this initiative to the residents’ education.

## EXAMPLE 2

In an effort to create a more compelling presentation, one of us took a previously well-received presentation on anterior segment ophthalmologic trauma and used educational theory and best practices to redesign it. This was done with the goals of improving participants’ learning and retention. Successful treatment of such traumas is largely dependent upon proper diagnosis based on pattern recognition, otherwise known as ***non-analytical clinical reasoning****.*^18^ Much like electrocardiograms,^19^ skin disorders,^20^ and radiographs,^21^ the more examples one sees, the more proficient one becomes. Expertise is based on building a repertoire of examples against which future experiences can be compared. Consistent with the conceptual basis of non-analytical reasoning, this initiative was designed to maximize participants’ cognitive schema/catalog development through ***spaced repetition*** involving specific anterior-segment injuries.^22^

Consistent with Kolb’s experiential learning theory,^16^ the structure of this experience was designed to provide staged, multifaceted, authentic learning experiences to maximize the breadth and depth of what was learned. Additional pedagogical principles were employed in the appropriate circumstances.

### Structure

#### I. Pre-Session

To activate learning, 10 pictures of specific pathological entities, each with clinically related questions, were sent to participants to complete and return one week prior to the presentation.

#### II. Presentation (one hour)

The objective was to create a clinical need to know followed by a case-based, concrete learning experience promoted through ***active learning techniques*** such as “think-pair-share” and “the one-minute paper.”^23^ This was carried out in a safe environment that facilitated participant decision- making and engagement in the experience. To view the presentation go to: https://chipcast.hosted.panopto.com/Panopto/Pages/Viewer.aspx?id=3a0780f2-0218-457b-a9f4-fb9c9ba493a4.

#### III. Workshop (one hour)

To build on prior experiences, small-group sessions were designed to promote learning from one another (***social learning theory***).^24^,^25^ Each group received several case scenarios with pictures and questions related to actual complex presentations. In a ***problem-based learning exercise***, each group worked through the cases provided, including (1) What are your initial concerns; (2) How will you proceed with evaluation; (3) What treatment options would you consider; and (4) What do you believe will be the final disposition of this patient?

#### IV. Post-session

Ten pictures involving eye trauma not previously seen, each with clinically relevant questions, were sent to participants to complete one week after the presentation. The purpose of this exercise was to provide additional examples for schema development and retention, not to test participants’ post-program knowledge.

Though there were generally multiple examples of most of the entities presented, high-risk presentations such as hyphemas (eight examples) and alkaline burns (five examples) were emphasized to maximize schema formation regarding these high-risk entities.

Participants’ post-program assessment of the experience was very positive (Kirkpatrick level 1), particularly in regard to learning clinically relevant information (Kirkpatrick level 2). Most reported specific, intended changes in their practice as a result of the experience (Kirkpatrick level 3). Though generally not substantive enough for scholarly work, such subjective “soft outcomes” are appropriate for evaluating and improving this type of experience. From a personal perspective, trying something new and experiencing an increased level of engagement from participants was both energizing and fulfilling as an educator.

## EXAMPLE 3

In 2014, our emergency medicine (EM) residency was faced with redesigning the pediatrics experience for our residents. The traditional model involving EM residents rotating “off-service” on pediatric inpatient wards had proved ineffective and unpopular. Residents voiced their concern that this learning environment did not resemble their intended practice. Significant time was spent in administrative and other tasks that were not germane to the practice of EM, and the educational benefit of the “ward rotation” was not perceived as being worthy of this opportunity’s cost. While there was certainly educational opportunity in the pediatric setting that could be valuable to EM residents, it was not clear how precisely to best achieve this benefit.

The decision was made to redesign the experience through the lens of ***situated learning*** – the conceptual framework in which learners are welcomed into a community of practice by participating in authentic work, connecting this experience with prior knowledge, and developing relationships with other professionals.^25^ By situating the learning experience in the authentic care of patients in the emergency department (ED), our hope was that residents would begin to understand the importance of pediatric care in the ED. Residents then followed up on all of their admitted patients during a mentored follow-up experience, rounding on the inpatient wards with a pediatric EM attending physician. In this manner, learners could further their understanding of both the longitudinal outcomes of hospitalization, and better recognize the role of the emergency physician (EP) in the continuum of pediatric care.

Framing our intervention in the situated learning framework, we used a common curriculum development rubric described by ***Kern et al***.^26^ to develop, design and study the new curriculum. Residents, faculty and alumni were surveyed to identify both general and specific needs related to pediatric care in the ED as well as to gain a perspective on the course of pediatric illness and injury. From this needs assessment, we developed goals and objectives for the new curriculum. These learning objectives then guided the selection of appropriate educational methods and strategies. Once implemented, the identified goals and objectives could then guide evaluation strategies by which to determine effectiveness of the curriculum. With a fresh set of intended outcomes focused specifically on application of pediatric knowledge for the EP, we had the means to evaluate whether the curriculum was achieving its goals.

Beyond designing a curriculum, however, the basis in conceptual frameworks and use of a systematic process to guide the development of goals and objectives had the welcome benefit of allowing our team to communicate our experiences to other educators – even those beyond our specialty. Speaking in the language of educational theory and outlining our goals, objectives and outcomes in the Kern framework, our work became more scholarly. We presented our findings at academic meetings and were able to publish our experience for others to read, critique, and build upon.^27^ To demonstrate the impact of our curriculum to the residency program and medical directorship, we focused on the learners› satisfaction (Kirkpatrick level 1) and on their perceptions regarding positive impact on clinical care in the ED (admittedly a lower-level learning outcome). Residents also reported self-assessed changes in knowledge (Kirkpatrick level 2). To demonstrate the impact of this initiative on behavior (Kirkpatrick level 3), we used ***self-reported, retrospective post-then-pre surveys***^28^ in addition to ***direct observation*** using a standardized assessment tool based on entrustable professional activities to provide a more objective, higher-level outcome. Without a firm basis in educational theory, this project would have remained a closed process, perhaps locally successful but surely not generalizable beyond our institution. Depending on the outcome, other curricula may require more objective data, such as tests of knowledge acquisition or changes in behavior, to reach the threshold of scholarly educational innovation. Incorporating education theory from the start, we could improve our product (the curriculum itself) and our scholarly impact.

Our hope is that these three examples will assist those who are interested in making their next educational intervention more evidence based. In addition to the references provided, a “toolbox” of potential resources has been included (Table 2) to facilitate the development of evidence based initiatives and achieving scholarly results.

## Figures and Tables

**Table 1:** Potential benefits to the use of evidence-based education.

Increased learning and improved retention from teaching activities. Results from questionnaires that accurately reflect what authors are attempting to understand. Curricula and programs that successfully meet their intended goals. Valid assessments of performance. Outcomes that demonstrate success to peers, administration, and the larger education community. The oppportunity to publish outcomes if the project is innovative and/or scholarly in its approach. Modeling an evidence-based approach for learners to emulate.

**Table 2:** Potential resources for developing evidence-based educational initiatives.

Resource	Website	Description
Best Evidence Medical Education (BEME)	https://www.bemecollaboration.org/Publications+Evidence+Based+Medical+Education/	The BEME Collaboration is an international group of individuals, universities and professional organizations committed to the development of evidence-informed education in the medical and health professions.
“Twelve tips” series	Website for Medical Teacher: https://www.tandfonline.com/loi/imte20	This series provides practical advice in the form of 12 short hints or tips on medical education topics of interest (ex. evaluating educational programs, flipping the classroom).
ALiEM Academic Primer Series	https://www.aliem.com/2017/06/academic-primer-series-curated-collections-for-educators/	Collection of nine narrative reviews on important medical education topics, highlighting the most important literature and their defined importance for junior educators and faculty developers.
Curated Collections for Educators	“Five Key Papers” series – published in both *West*JEM and *Cureus*	This series provides the five most important papers on specific topics of importance in medical education. Topic examples include educational scholarship in junior academic faculty and digital scholarship.
International Clinician Educators (ICE) Blog	https://icenetblog.royalcollege.ca	This blog promotes discussion among clinician educators from around the world, archiving a variety of education resources.
Key Literature in Medical Education (KeyLIME) podcast	https://icenetblog.royalcollege.ca	This is a weekly podcast produced by the Royal College of Physicians and Surgeons of Canada that provides a summary and analysis of a medical education article in under 30 minutes.
AMEE Guides	https://amee.org/publications/amee-guides	AMEE Guides cover topical issues in medical and healthcare professions education and provide information, practical advice and support.
ALiEM Education Theory Made Practical eBooks	https://www.aliem.com/2017/08/education-theory-made-practical-volume-1/	This is a free, peer-reviewed eBook that explains educational theories and how they can be integrated into educational practice.

*ALiEM*, Academic Life in Emergency Medicine; *WestJEM*, Western Journal of Emergency Medicine; *AMEE*, Association for Medical Education in Europe.
